# Src dependency of the regulation of LTP by alternative splicing of *GRIN1* exon 5

**DOI:** 10.1098/rstb.2023.0236

**Published:** 2024-07-29

**Authors:** Hongbin Li, Vishaal Rajani, Ameet S. Sengar, Michael W. Salter

**Affiliations:** ^1^ Program in Neurosciences & Mental Health, The Hospital for Sick Children, Toronto, ON M5G 1X8, Canada; ^2^ Department of Physiology, University of Toronto, Toronto, ON M5S 1A8, Canada

**Keywords:** hippocampus, NMDA receptors, AMPA receptors, synaptic plasticity

## Abstract

Alternative splicing of *Grin1* exon 5 regulates induction of long-term potentiation (LTP) at Schaffer collateral-CA1 synapses: LTP in mice lacking the GluN1 exon 5-encoded N1 cassette (GluN1a mice) is significantly increased compared with that in mice compulsorily expressing this exon (GluN1b mice). The mechanism underlying this difference is unknown. Here, we report that blocking the non-receptor tyrosine kinase Src prevents induction of LTP in GluN1a mice but not in GluN1b. We find that activating Src enhances pharmacologically isolated synaptic *N*-methyl-d-aspartate receptor (NMDAR) currents in GluN1a mice but not in GluN1b. Moreover, we observe that Src activation increases the α-amino-3-hydroxy-5-methyl-4-isoxazolepropionic acid (AMPA) receptor component of Schaffer collateral-evoked excitatory post-synaptic potentials in GluN1a mice, but this increase is prevented by blocking NMDARs. We conclude that at these synapses, NMDARs in GluN1a mice are subject to upregulation by Src that mediates induction of LTP, whereas NMDARs in GluN1b mice are not regulated by Src, leading to Src-resistance of LTP. Thus, we have uncovered that a key regulatory mechanism for synaptic potentiation is gated by differential splicing of exon 5 of *Grin1*.

This article is part of a discussion meeting issue ‘Long-term potentiation: 50 years on’.

## 1. Introduction

*N*-methyl-d-aspartate receptors (NMDARs), a major subtype of ionotropic glutamate receptor, are critical for physiological function and plasticity across the mammalian CNS, and dysfunction of NMDARs underlies a host of CNS disorders [1]. These receptors are increasingly understood to signal not only by opening the channel pore (i.e. ionotropically) but also without pore opening [2,3] (i.e. non-ionotropically). Canonical NMDARs are multiprotein complexes at the core of which is the pore-forming and ligand-binding heterotetramer composed of two GluN1 and two GluN2 subunits, which assemble as dimers of dimers together with signalling, scaffolding and regulatory proteins [1,4]. NMDARs are co-receptors for glutamate and glycine (or d-serine), with neither agonist alone sufficient to gate the pore open. The binding sites for glutamate are on each of the GluN2 subunits and those for glycine are on the GluN1 subunits [1]. In some cases, one or both of the dimers may include GluN3 rather than GluN2, with GluN1-GluN2 receptors being excitatory gylcine receptors [5].

Post-synaptic responses at glutamatergic synapses typically have two components with the faster one by AMPA receptors (AMPARs) and the slower one mediated by NMDARs. At the basal physiological resting membrane potential of most CNS neurons, NMDAR current flow is impeded by Mg^2+^ blockage of the pore, and basal fast excitatory synaptic transmission is thus dominated by AMPARs. Mg^2+^ blockage of NMDARs is highly voltage-dependent, with the inhibition of current flow by Mg^2+^ producing a region of ‘negative slope conductance’ in the current–voltage relationship [6]. At the resting-membrane potential, current flow is suppressed (often erroneously said to be ‘blocked’) but at sufficiently depolarized membrane potentials the Mg^2+^ impediment is relieved, allowing unimpeded flow of Na^+^, K^+^ and Ca^2+^ ions. As basal extracellular glycine or d-serine is typically sufficient for gating of NMDARs, the characteristic voltage-dependency of the receptors allows the current flow to be highly sensitive to the coincidental pre-synaptic release of glutamate and post-synaptic depolarization. This characteristic is well-suited to the receptors functioning as triggers initiating persistent changes in synaptic efficacy. Indeed, glutamatergic synapses show a high degree of bidirectional plasticity [7]. Here, we focus on the role of GluN1 splice variants in regulating the lasting enhancement of synaptic transmission—long-term potentiation (LTP) at Schaffer collateral synapses on CA1 neurons in the hippocampus—which is the predominant cellular model of learning and memory [8]. Induction of LTP at these synapses depends upon activating NMDARs, whereas LTP is expressed as an increased number and/or gating of post-synaptic AMPARs [7,9].

**Figure 1.**
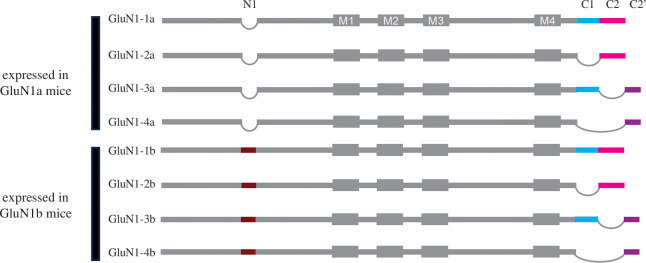
The eight GluN1 isoforms generated by alternative splicing of *Grin1*. The four cassettes called N1, C1, C2 and C2’ are generated from exons 5, 21, 22 or 22’, respectively. The transmembrane (TM) helices are shown as M1, M3 and M4. M2 indicates the membrane re-entrant loop. All eight isoforms are expressed in WT mice. The four GluN1 isoforms expressed in GluN1a mice and the four expressed in GluN1b mice are indicated. The illustration is not to scale. Modified from [11], with permission.

GluN1 is encoded by a single gene, *Grin1*, with eight splice variants, whereas the four GluN2 subunits are encoded by separate *Grin2A-D* genes [1,10]. Each of the GluN1 variants together with any of the GluN2 subunits is competent to form functional heterotetramers. Transcription and processing of *Grin1* produces the mRNA variants through inclusion or exclusion of the N-terminal-encoding exon 5 and of the C-terminal exons 21, 22 or 22’ (figure 1). The eight *Grin1* splice variants are expressed in the CNS, with the relative expression of each varying developmentally, regionally and by cell type [12,13]. In contrast to *Grin1*, each *Grin2* gene produces a single mRNA species, except for GluN2A in primates, where a short splice isoform GluN2A-S lacking 183 amino acids has recently been identified [14]. The common wisdom, as exemplified by the enormous amount of work developing GluN2 subunit-selective agents [1], is that it is differences imparted by varying compositions of the GluN2 subunits that underlie the great diversity of CNS functions and dysfunctions controlled by NMDARs. Although differential splicing of *Grin1* was identified quickly after the subunit was cloned about 30 years ago [15,16], and despite greater diversity in GluN1 than in GluN2 (i.e. eight GluN1s versus four GluN2s), the *in vivo* consequences of diversity in GluN1 subunits have not been explored until relatively recently.

The N-terminal alternatively spliced exon—exon 5—encodes the 21 amino acid N1 cassette (figure 1). The four GluN1 isoforms lacking the N1 cassette are designated ‘a’, as the first cloned GluN1 lacked exon 5 [15,17], whereas the four isoforms containing N1 are designated ‘b’. N1 does not affect crucial ionotropic characteristics of NMDARs such as glycine and glutamate potency, and voltage-dependent blockage by Mg^2+^ [1,16]. However, N1 does affect allosteric regulation by H^+^, Zn^2+^, Mg^2+^ [18] and extracellular polyamines [1], channel deactivation [1,19] and also glycine-site mediated non-ionotropic signalling [20].

The C-terminal exons 21, 22, 22’ are alternatively spliced leading to four variants named 1-1 to 1-4 in which are encoded C1, C2 and C2’ cassettes. Deleting exon 22 removes the stop codon, resulting in the inclusion of C2’. These splice variants do differentially affect membrane trafficking and receptor clustering [21,22]. The C1 cassette contains three serine phosphorylations that regulate subcellular localization [22]. Serines 890 and 896 are phosphorylated by protein kinase C and serine 897 by protein kinase A [23]. Clustering of NMDARs is impaired by phosphorylation of serine 890, and receptor cell surface expression is increased by phosphorylating serines 896 and 897.

The N1 cassette shows 100% amino acid sequence conservation across a wide range of vertebrates [11], and no sequence changes in exon 5 are found in the known variants of human *GRIN1* [11,19]. By contrast, in exons 21, 22 and 22’, there is considerable cross-species variability and numerous non-synonymous variations in humans [11]. The lack of variants in the sequence of the N1 cassette indicates that this sequence is under strong evolutionary selection pressure [24,25]. In the CNS, both GluN1a and GluN1b isoforms are expressed, indicating that there is pressure to retain not just one but both variants, i.e. that each has critical function(s). Importantly, the distribution of the expression of each of the isoforms is not uniform across regions or neuronal cell types in the CNS [12,13]. In the brain as a whole, GluN1a isoforms are expressed at a higher level than GluN1b isoforms. However, some neuronal subtypes express relatively more GluN1b isoforms than GluN1a [26]. The differing relative levels of expression of GluN1a versus GluN1b isoforms between regions and cell types provides the basis for the concept that alternative splicing of exon 5 of GluN1 may confer novel mechanisms on NMDARs to regulate diverse functions in different regions and cell types in the CNS.

To characterize the *in vivo* functions of exon 5 splicing, we generated mice lacking GluN1 exon 5 (GluN1a) and mice compulsorily expressing this exon (GluN1b) [11]. We found that both GluN1a and GluN1b mice develop normally, not differing from development in the wild type (WT). As a first step in exploring physiological roles of exon 5 splicing, we looked at the CA1 region of the hippocampus where the 1a predominance would be accentuated in GluN1a mice, and eliminated in GluN1b mice. We found that the mice do not differ from each other, or from WT, in basal synaptic transmission, synaptic input–output or short-term synaptic plasticity. Nor do they differ in agonist potency or Mg^2+^ blockade. Also, long-term depression is not dissimilar across the three genotypes. Given this degree of similarity, it was unexpected to discover that the magnitude of LTP in CA1 is significantly less in GluN1b mice as compared with that in GluN1a mice [11]. The greater magnitude of LTP in GluN1a mice than in GluN1b was found with both of the LTP-inducing stimulation protocols tested—theta-burst stimulation (TBS) and 20 Hz [11].

As we found differential splicing of *Grin1* exon 5 altered NMDAR-dependent LTP in the hippocampus, we wondered whether alternative splicing of this exon might affect NMDAR-dependent learning and memory [27]. To this end, we tested mice of the three genotypes using the Morris water maze (MWM) task, which is well-established as a test of NMDAR-dependent learning and memory [28]. We found that GluN1a mice perform better than GluN1b mice in this test: GluN1a mice learn more quickly and have significantly better spatial memory performance on the MWM test than GluN1b mice [11]. Also, GluN1b mice had significantly worse performance as compared with WT on the final two training days and on the 2 h probe after the final training day. Importantly, there were no differences in the relative expression of the *Grin1* C-tail exons or of *Grin2A-D* [11]. Thus, we discovered important physiological functions that are differentially controlled by alternative splicing of exon 5 of *Grin1*. Our findings indicate that synaptic plasticity, learning and memory are not solely regulated by the type of GluN2 subunit.

Our findings raise the question of how the presence of the N1 cassette leads to the reduction in the magnitude of LTP, and in learning and memory performance. We found no differentially expressed genes between the GluN1a, GluN1b or WT mice in RNAseq from the forebrain [11], indicating that if there are any changes produced by the sole expression of the N1 cassette they do not cause the brain to alter its transcriptome. Also, the level of expression of the C-terminal *Grin1* exons was not different in GluN1a as compared with GluN1b mice. Nor did the expression of *Grin2A* or *Grin2B*, the two major partners of GluN1, differ between the GluN1a and GluN1b mice. Functionally, there were no differences in basal synaptic transmission, in NMDAR-to-AMPAR ratio, nor in the potency or voltage-dependence of NMDAR excitatory post-synaptic currents (EPSCs). Thus, the N1 cassette appeared to have no observable change in the assembly, trafficking, stability or basal activity of synaptic NMDARs that might otherwise have been able to account for the differences in LTP or learning and memory.

Induction of LTP has been found to be mediated by relief of Mg^2+^ blockade of NMDAR channels [8], and also by biochemical amplification of NMDAR currents by the non-receptor tyrosine kinase Src [29–33], both processes driving influx of Ca^2+^ that leads to increased AMPAR-mediated transmission [30,34]. A body of evidence [32,34] indicates that upon LTP-inducing stimulation, Src activity is rapidly increased [30] by a highly localized signalling network [35] involving its direct regulators Csk [36] and PTPα [37]; with CAKβ/Pyk2 further upstream [38]. Activation of Src not only directly increases NMDAR activity but also sensitizes the receptors to further increased activity by increasing intracellular [Na^+^] [39]. Coincident with the activation of Src is the suppression of striatal enriched phosphatase (STEP) [40], which opposes the action of Src on NMDARs [41]. These signalling events come together to drive unimpeded Src-mediated amplification of synaptic NMDAR currents.

Src is a component of the NMDAR complex [4,35] that, when activated, increases NMDAR currents by increasing the open probability and mean open time in NMDAR superclusters [29]. It has been found that Src is anchored in the NMDAR complex by an interaction between a stretch of amino acids (40–58) in its unique domain that binds to the protein ND2 [42] through an intracellular hydrophilic region between transmembrane (TM) regions 10 and 11 [43]. ND2 itself is held in the NMDAR complex by an interaction between a deep groove, lined by TMs 1, 5, 8 and 11, with M4 of GluN1 [43]. Here, we investigated the possibility that there might be differences in Src-mediated upregulation of GluN1a versus GluN1b NMDARs and that this might underlie the difference in the magnitude of LTP at Schaffer collateral synapses in these mice.

## 2. Results and discussion

### (a) Differential effects of Src on LTP and NMDARs in GluN1a versus GluN1b

As LTP induction involves relief of Mg^2+^ blockade and Src upregulation of NMDAR currents [29–33], it is possible that the difference in LTP between GluN1a and GluN1b might stem from differences in Mg^2+^ blockade or differences in Src-mediated upregulation of synaptic NMDAR responses. However, we found that Mg^2+^ blockage in GluN1b mice does not differ from that in GluN1a mice [11]. With respect to Src-mediated upregulation, we have found previously that there is a depolarization evoked by TBS, referred to as the ‘burst’ excitatory post-synaptic potentials (EPSPs), which is mediated by enhancement of NMDARs through Src kinase [44]. This enhancement, we have reasoned, is important for the induction of LTP [34], and may be used as a readout of rapid upregulation of synaptic NMDAR currents by Src. In comparing the burst EPSPs in CA1 pyramidal neurons from GluN1a versus GluN1b mice, we have found that in GluN1b neurons burst EPSPs are significantly smaller than those in GluN1a neurons [11]. From the smaller burst EPSPs in GluN1b neurons, we considered the possibility that impairment of Src-mediated enhancement of NMDAR currents may underlie the lower magnitude LTP in the GluN1b mice as compared with GluN1a mice.

**Figure 2.**
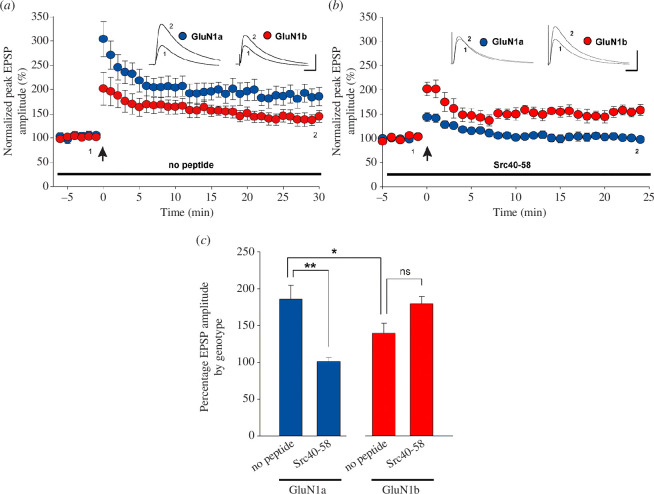
Src inhibitory peptide, Src40-58, blocks LTP induction in GluN1a but not in GluN1b (*a*). Scatter plot of the normalized peak EPSP amplitude over time of recordings from GluN1a neurons (*n* = 5 neurons from three animals) and GluN1b neurons (*n* = 10 neurons from three animals). For these recordings, no peptide was included in the intracellular solution. Inset traces show a representative average of five EPSPs at the times indicated by 1 and 2 in the main plot. TBS was delivered at the time indicated by the arrow. (*b*). Scatter plot of the normalized peak EPSP amplitude over time for recordings from GluN1a neurons (*n* = 7 neurons from six animals) or GluN1b neurons (*n* = 6 neurons from four animals). For these recordings, Src40-58 peptide was included in the intracellular solution. Inset traces show representative averages of five EPSPs at the times indicated by 1 and 2 in the main plot. TBS was delivered at the time indicated by the arrow. (*c*) Histograms of average normalized EPSPs peak amplitude for the GluN1a and the GluN1b neurons 25–30 min after TBS. The treatment is indicated below each bar. *p < 0.05; **p < 0.01; ns, *p* > 0.05. All experiments shown in this figure were done with whole-cell patch recording in current-clamp mode. EPSPs were evoked by stimulating Schaffer collateral inputs to CA1. Scale bars in *a*, *b*, 10 mV; 50 ms.

We thus tested the possibility that GluN1a and GluN1b may differ in Src-dependency of LTP induction. During whole-cell current-clamp recordings from CA1 pyramidal neurons, we intracellularly administered the peptide Src40-58, which blocks the interaction between Src and its anchoring protein ND2 and displaces Src from the NMDAR complex, making the kinase unavailable to enhance NMDAR currents [29,35]. Thereby, Src40-58 prevents induction of LTP in WT animals [29,35,41]. Here, we found in no peptide control experiments (figure 2*a*) that LTP was induced in CA1 neurons of both GluN1a and GluN1b mice, with the magnitude in GluN1a greater than that in GluN1b (figure 2*a*,*c*). Intracellularly administering Src40-58 prevented induction of LTP in GluN1a mice (figure 2*b*,*c*). By contrast, introducing Src40-58 into GluN1b neurons did not prevent LTP induction (figure 2*b*) nor did it affect LTP magnitude (figure 2*c*).

**Figure 3.**
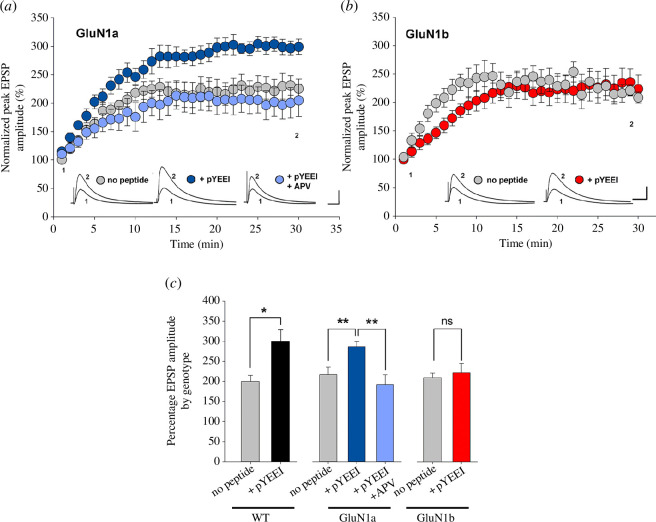
Src family kinase activating peptide, pYEEI, enhances EPSPs in GluN1a but not in GluN1b neurons (*a*). Scatter plot of normalized peak EPSPs from GluN1a neurons. The intracellular solution had no pYEEI peptide added (*n* = 6 neurons from three animals), or pYEEI included without (*n* = 7 neurons from three animals) or with bath-applied (2R)-amino-5-phosphonovaleric acid (APV) (*n* = 6 neurons from three animals). (*b*). Scatter plot of normalized peak EPSPs from GluN1b neurons. The intracellular solution had no pYEEI peptide added (*n* = 6 neurons from four animals), pYEEI included (*n* = 7 neurons from four animals). (*c*) Histograms of average normalized EPSPs peak amplitude for WT (*n* = 6 neurons from three animals (with peptide) and 6 neurons from four animals (no peptide)), the GluN1a or the GluN1b neurons with treatment indicated below each bar. The average amplitude was calculated for 25–30 min after breakthrough. The run-up of the peak amplitude with no added pYEEI was not statistically significantly different across the three genotypes. *p < 0.05; **p < 0.01; ns, *p* > 0.05. All experiments shown in this figure were done with whole-cell patch recording in current-clamp mode. EPSPs were evoked by stimulating Schaffer collateral inputs to CA1. In all panels, response amplitudes were normalized to those evoked in the first 2 min after patch breakthrough. Inset traces in *a* and *b* show a representative average of 10 EPSPs recorded at the beginning (1) and the end (2) of the 30 min period shown. Scale bars in *a*, *b*, 10 mV; 50 ms.

We examined the effect of the phosphopeptide EPQ(pY)EEIPIA (abbreviated pYEEI), which activates Src family kinases by de-repressing the inhibitory intramolecular binding of pY427 to the SH2 domain [45]. pYEEI has been shown in the WT to increase AMPAR-mediated synaptic responses indirectly through a signalling cascade that depends upon upregulating NMDARs and the consequential increase in intracellular [Ca^2+^] [29,38,41]. Here, we made whole-cell recordings from CA1 neurons using a low Ca^2+^ buffering intracellular solution in order to permit Ca^2+^-mediated processes. During recordings, because we were administering pYEEI intracellularly through the patch pipette, we established the initial EPSP peak amplitude as the average of the responses during the first 2 min after patch breakthrough. In recordings without peptide from GluN1a and from GluN1b neurons, we observed a gradual, consistent ‘run-up’ of the response amplitude during the first 10–12 min, reaching a stable level thereafter (figure 3). During recordings from GluN1a neurons in which pYEEI was administered through the patch pipette, we found that the rate of the initial increase in EPSP amplitude and the final, stable level were greater than during no peptide recordings (figure 3*a*,*c*). By contrast, in GluN1b neurons recorded with pYEEI administered intracellularly neither the rate of run-up nor the final level were different from no peptide recordings (figure 3*b*,*c*). We conclude from these findings that pYEEI caused an increase in EPSP amplitude in GluN1a neurons but not in GluN1b. Moreover, we found that the increase in EPSP amplitude in GluN1a neurons was prevented by blocking NMDARs with the antagonist (2R)-amino-5-phosphonovaleric acid (APV) (figure 3*a*,*c*), indicating that NMDAR activity is required for the increased EPSP amplitude caused by pYEEI.

**Figure 4.**
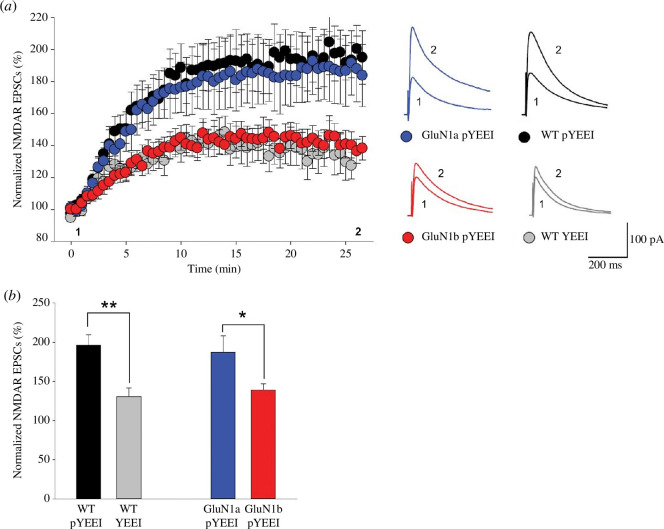
Effects of Src family kinase activation on pharmacologically isolated NMDAR EPSCs. (*a*). Scatter plot of normalized peak amplitude of NMDAR EPSCs from WT neurons in which YEEI control peptide (*n* = 6 neurons from two animals) was included in the intracellular solution, or from WT (*n* = 12 neurons from six animals), GluN1a (*n* = 6 neurons from six animals) or GluN1b (*n* = 9 neurons from six animals) neurons in which the intracellular solution contained pYEEI. Representative average traces of 10 EPSCs at times indicated by 1 and 2 are shown to the right for each of the four conditions. Recordings were done with whole-cell patch recording in voltage-clamp mode. Scale bar 100 pA; 200 ms. (*b*). Histograms of average normalized NMDAR EPSC peak amplitude for the genotypes and treatments indicated below each bar. In both panels, response amplitudes were normalized to those evoked in the first two minutes after patch breakthrough. The average amplitude was calculated as the last 5 min of the recordings. *p<0.05, **p<0.01.

Our findings that in GluN1a neurons, but not in GluN1b, Src40-58 inhibits LTP induction and pYEEI causes an (2R)-amino-5-phosphonovaleric acid (APV)-sensitive enhancement of EPSPs are evidence that Src enhancement of NMDAR currents, which is normally required for the induction of LTP in WT, may also be present in GluN1a neurons but may be lacking in GluN1b neurons. To test this, we therefore examined pharmacologically isolated NMDAR EPSCs, with AMPARs blocked by CNQX and with high intracellular Ca^2+^ buffering. During recordings from CA1 neurons of WT mice, we found that NMDAR EPSCs were increased by intracellularly applying pYEEI as compared with WT neurons in which the non-phosphopeptide YEEI, which does not activate Src kinases [45], was applied (figure 4), as we have reported previously [29,38,41]. During recordings from neurons of GluN1a mice, applying pYEEI increased NMDAR EPSCs to a level indistinguishable from that of the WT (figure 4). However, in recordings from GluN1b CA1 neurons, pYEEI caused no increase in NMDAR EPSCs comparable to that in WT or GluN1a neurons. Rather the run up of EPSC amplitude was similar to that seen in recordings in which YEEI was administered.

Together, we take our findings as evidence that in GluN1a, as in WT, CA1 neurons AMPAR EPSPs are increased as a consequence of the Src-mediated enhancement of NMDAR currents. We note that in WT CA1 neurons, the long-lasting enhancement of EPSPs induced by activating Src either by pYEEI [30] or by endogenous upstream Src activators [36,38] occludes the induction of LTP by tetanic or TBS stimulation of Schaffer collaterals. Thus, we conclude that as in those experiments, the persistent increase of EPSPs by pYEEI that we report here in GluN1a CA1 neurons is a form of AMPAR-LTP induced as a consequence of Src enhancement of NMDAR currents.

In contrast to LTP in GluN1a CA1 neurons, the LTP in GluN1b neurons was unaffected by Src40-58 and therefore appears to not require Src. There is evidence that another Src family kinase, Fyn, may increase NMDA-evoked currents [46,47] and NMDAR EPSCs, particularly in the spinal cord [48], and Fyn regulation of LTP has been observed in the hippocampus [49]. Fyn upregulation of NMDARs is insensitive to Src40–58 [49,50], and thus it is conceivable that Fyn might substitute for Src in GluN1b neurons. However, if this were the case one would have expected to find that NMDAR EPSCs in GluN1b CA1 neurons would be increased by pYEEI, which activates all SFKs [35]. Therefore, it seems unlikely that in GluN1b mice, Fyn is substituting for Src in induction of LTP. By similar reasoning, the other Src kinases expressed in the mammalian CNS [35]—Yes, Lck and Lyn—are unlikely to mediate LTP induction in GluN1b mice, especially because, unlike Src and Fyn, none of these is found in the post-synaptic density [51,52].

### (b) Src enhancement of NMDARs is required for CA3–CA1 LTP only for GluN1a-containing NMDARs

From previous work by our group and others, we have proposed that relief of Mg^2+^ blockade alone may not be sufficient but rather an additional mechanism to boost NMDAR currents, by stimulating signalling that activates Src within the NMDAR complex is also required for LTP induction [32,34]. Upstream activators of Src that are required for LTP have been identified, the effects of each of which have been found—like direct activation of Src by pYEEI—to act via increasing NMDAR currents. Intracellular stimulation of the kinase CAKβ/Pyk2 causes a Src-dependent potentiation of NMDAR currents and occludes NMDAR-dependent LTP, while a dominant negative CAKβ/Pyk2 prevents LTP induction [38]. Blocking the endogenous Src activator, PTPα suppresses Src potentiation of NMDAR currents and induction of LTP in hippocampal neurons [37]. Upstream inhibitors also affect Src-dependent LTP [34]. Disrupting Csk, the tyrosine kinase that phosphorylates the C-terminal autoinhibitory site on Src, with a function-blocking antibody increases NMDAR currents and induces LTP [36]. In addition, in CA1, the phosphotyrosine phosphatase STEP opposes the increase in NMDAR activity produced by Src, preventing the potentiation of NMDARs and acting tonically as the main brake on LTP induction [41]. Under basal conditions in WT, the activity of STEP dominates that of Src but STEP is unable to keep up with the increase in Src activity caused by LTP-inducing stimulation [41]. Engaging the Src signalling network as a result of synaptic activity provides a biochemical form of coincidence detection, which works together with that produced by post-synaptic depolarization. One may wonder whether biochemical signalling can work sufficiently quickly to be involved in LTP induction. We note that the key enzyme—Src—is strategically present in the NMDAR complex, in immediate proximity to their substrates, the GluN2 NMDAR subunit proteins. The catalytic turnover rate of kinases is thousands of cycles per second [53], so more than ample to rapidly produce the necessary phosphorylation, especially considering that LTP-inducing stimulation protocols are delivered over several seconds. Empirically, there is no Src-dependency to basal NMDAR currents owing to the opposition by STEP [38]. But Src-dependency of the amplification of the NMDAR component of the TBS-induced burst EPSP is apparent within the response to the initial theta burst, i.e. within 40 min after the very first stimulus [44]. This concept is consistent with findings that LTP-inducing stimulation is found to increase Src activity, assayed biochemically from tissue punches of CA1 stratum radiatum, as fast as it can be measured [44].

**Figure 5.**
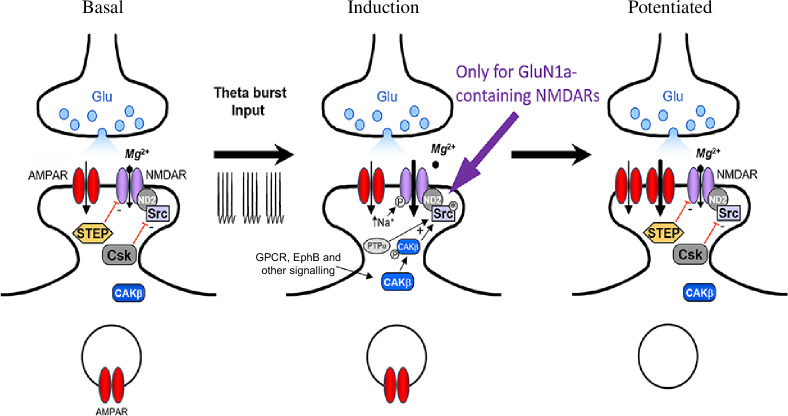
Model for Src mediation of LTP induction in GluN1a mice. Under basal conditions (left), synaptic NMDAR EPSCs are suppressed by Mg^2+^ reduction of current flow through the channel and by biochemical downregulation of channel activity, directly by STEP and indirectly through Csk inhibition of Src. During TBS, NMDAR EPSCs are amplified by: (i) depolarization-induced relief of Mg^2+^ inhibition, (ii) increased gating of GluN1a-containing NMDARs through Src activation (Src*) by PTPα and activated CAKβ (P-CAKβ), which together surpass the action of STEP, and (iii) the resultant sensitization of NMDARs to increased intracellular [Na^+^]. The electrical and biochemical amplification of NMDAR EPSCs boosts entry of Ca^2+^, leading to calmodulin-CaMKII-mediated (not illustrated) expression of LTP by increasing the number and/or activity of postsynaptic AMPARs. GPCR, G-protein-coupled receptor. Modified from [34].

Putting these electrical and biochemical signalling events together, we have developed a framework for the induction of LTP at CA3–CA1 synapses [32,34]. The evidence supporting this framework was acquired from studies done using WT animals. Our new findings, reported here, require revising this framework, such that the Src-dependent form of LTP at these synapses applies to NMDARs lacking the N1 cassette, GluN1a receptors, but not to those containing the N1 cassette, GluN1b receptors (figure 5). The readily apparent explanation as to how the Src-dependency was ever elucidated is that in CA1, the amount of NMDARs lacking N1 far exceeds that of receptors containing N1.

Our current findings raise the question of why LTP in GluN1b neurons apparently independent of Src kinase? As Src is present in the NMDAR complex in the WT [4,29], which is dominated by GluN1a-containing receptors, the simplest possibility is that Src is absent from NMDAR complexes composed of N1-containing GluN1s. The N1 cassette alters the local architecture of the heterotetrameric assembly, creating contacts with the ligand-binding domains of the subunits that are absent in GluN1a-containing receptors [54], thereby altering the structure in extracellular regions of the NMDAR heterotetramer [54,55]. It is possible that the effect of the extracellular structural alteration is transmitted across the cell membrane. Conformational changes in the intracellular C-terminal domain of NMDARs are known to be transmitted across the membrane following changes in the extracellular domain [56] and such extracellular-induced TM-transduced changes in intracellular domains are commonplace for G-protein-coupled receptors [57]. Thus, it is not inconceivable that the extracellular N1-cassette induces alterations of intracellular protein–protein interactions, in particular, the interaction that anchors Src in the NMDAR complex. Thus, either directly or indirectly, the N1 cassette may disrupt the computational logic of the NMDAR complex. That is to say, it remains to be investigated whether the N1 cassettes provoke structural changes transmitted within or across the membrane that affect the binding of Src to its anchoring protein ND2 [42], or of the binding of ND2 to GluN1 [43], such that with GluN1b-containing NMDARs Src is not in the NMDAR complex of GluN1b receptors.

Alternatively, or in addition, it is possible that even if Src is present in GluN1b-containing NMDAR complexes, its phosphorylation of NMDARs might be undone rapidly by excessive activity of STEP. Conversely, it is conceivable that Src is already maximally phosphorylating NMDARs in GluN1b, which partially occludes further enhancement of the post-synaptic responses by LTP-inducing stimulation of Schaffer collaterals. For example, as the receptor tyrosine kinase ErbB4 is known to cause ongoing, partial suppression of LTP induction by tonically suppressing Src activity [44,58] and ErbB4 is in the NMDAR complex through binding to PSD-95 [59], it is possible that GluN1b-containing NMDAR complexes lack ErbB4, which would result in the loss of tonic Src inhibition and occlusion of the potentiation by pYEEI or by stimulating Schaffer collaterals. Similar logic can be applied to the other upstream activating and inhibiting pathways for Src, which means that several types of future experiments will be required to sort out the apparent lack of Src upregulation of GluN1b-containing NMDARs.

### (c) Implications for GluN1b dominant neurons

Schaffer collaterals make excitatory synapses not only on pyramidal neurons but also onto inhibitory interneurons in CA1. Hippocampal interneurons, particularly the parvalbumin (PV)-expressing subtype, have much higher relative levels of exon 5-containing *Grin1* transcripts than do excitatory, pyramidal, neurons [26]. And, there is evidence that Schaffer collateral-evoked NMDAR EPSCs in inhibitory neurons are mediated by receptors in which the GluN1 subunits contain the N1 cassette [20]. Thus, a testable prediction from the above findings is that synaptic NMDAR currents in PV interneurons, and perhaps other inhibitory interneurons, are not subject to regulation by Src. Whether such lack of Src regulation of synaptic NMDARs, if found, contributes to LTP at Schaffer collateral-interneuron synapses [60] remains to be determined. Interneuron network-driven gamma oscillations are critical for memory formation and information processing [61], and it is conceivable that the expression of GluN1b NMDARs may be relevant to this important function of interneurons. Our findings open up the possibility that it is the specific expression of GluN1b NMDARs that is critical for synaptic plasticity and functions of brain regions where high-level exon 5-containing *Grin1* mRNA is expressed—thalamus, CA3 hippocampus and granule cells in the cerebellum [62].

### (d) Evolutionary conservation of exon 5 and its alternative splicing

Differential exon splicing provides a significant source for structural and functional diversity of proteins across and within species [63]. Exon 5 of *GRIN1* is highly conserved across vertebrates and the N1 sequence itself shows 100% primary sequence identity across species to bony fishes. Strikingly, single nucleotide polymorphisms (SNPs) in exon 5 are absent from genomic databases of humans [11]. The short length of exon 5 (63 bases) cannot account for the lack of SNPs in this exon because, in contrast to exon 5, SNPs are documented for *GRIN1* exon 22’, which is similar in length to exon 5. One interpretation would be that the N1 sequence imparts a critical property, or properties, to GluN1 that causes high evolutionary selection pressure to maintain the identity of its amino acid sequence across a range of species [64]. However, the exon 5 coding sequencing, and thus the presence of the N1 cassette in the GluN1 protein, is not required for normal development, growth or reproduction at least in the mouse [11]. Our findings raise the possibility that it is the mixture of N1-containing and Src-regulated N1-lacking GluN1s, along with differing proportions of these in different regions and cell types in the CNS, that provides the powerful survival advantage. We speculate that the mixture allows for tuning synaptic potentiation to an optimal level within the ideal dynamic range suited to the specific function of each brain region and cell type that in the natural environment provides a highly selective survival advantage.

### (e) Alternative splicing of exon 5 in *GRIN1* in stroke and neurodegeneration

In addition to regulating LTP and learning and memory, alternative splicing of exon 5 has been found to control the internalization of NMDARs that is primed non-ionotropically by stimulating the glycine site, independent of the glutamate site [65], of the receptor. Our present results raise the possibility that Src may mediate or regulate the priming or internalization that is seen with GluN1a-containing NMDARs but that is absent with GluN1b-containing receptors [20]. Glycine has been found to be strongly neuroprotective in *in vitro* [66] and *in vivo* [67–70] models of ischemic stroke. A growing body of evidence suggests that neuroprotection is mediated by non-ionotropic NMDAR function [66,67,70] (i.e. by glycine-primed NMDAR internalization). These studies have been done using WT animals. As GluN1a-containing NMDARs show glycine-primed internalization but GluN1b-containing receptors do not show this, it is likely that glycine-induced neuroprotection will be enhanced in GluN1a mice but will be lacking in GluN1b mice.

It is conceivable, as well, that alternative splicing of exon 5 controls pathological neuronal cell death more broadly, in particular in neurodegeneration. From studies on GluN1 splicing in post-mortem brains of individuals with Alzheimer’s disease (AD), it has been found that expression of exon 5-containing isoforms is greatly reduced in the brain areas showing the most significant pathology—hippocampus, cingulate cortex and superior temporal cortex [71,72]. But, exon 5-containing isoform levels are not affected in areas lacking AD pathology—motor and occipital cortices. Also, younger AD cases, which would have had greater disease severity, show a larger disparity in GluN1b : GluN1a ratio than in older cases or controls. One interpretation of these findings is that neurons preferentially expressing GluN1b-containing versus GluN1a-containing NMDARs may be selectively vulnerable to neurotoxicity in AD.

## 3. Concluding remarks

Here, we have shown that in GluN1a mice LTP at Schaffer collateral synapses depends upon Src upregulation of NMDARs. But, the induction of LTP in GluN1b mice is independent of Src. Thus, we have uncovered that a key regulatory mechanism for synaptic potentiation, Src upregulation of NMDARs, is gated by differential splicing of exon 5 of Grin1. As alternative splicing of exon 5 varies in various brain regions and cell types, Src may differentially regulate distinct NMDAR-dependent physiological processes and pathological disorders.

## 4. Methods

Experiments were done in accordance with policies and requirements of the Hospital for Sick Children Animal Care Committee and the Canadian Council on Animal Care. Methods for brain slice preparation and patch-clamp electrophysiology, described in brief below, were used as described in detail elsewhere [11,20].

### (a) Animals

Homozygous GluN1a and GluN1b mice were generated and used as described previously [11]. The experimenter was blinded to the genotypes of the animals. Experiments included animals of each sex.

### (b) Brain slice preparation

Mice aged 25–30 days were anaesthetized by intraperitoneal (i.p.) injection of 20% urethane (w/v). We prepared parasagittal hippocampal slices (300 μm) in ice-cold ACSF, composed of (in mM) 124 NaCl, 2.5 KCl, 1.25 NaH_2_PO_4_, 2 MgCl_2_, 2 CaCl_2_, 26 NaHCO_3_ and 11 d-glucose saturated with 95% O_2_ (balanced with 5% CO_2_) (pH 7.40, osmolarity 305). We placed the slices in a holding chamber (30°C) for 40 min and then allowed them to passively cool to room temperature (22°C) for ≥30 min before recording.

### (c) *Ex vivo* brain slice electrophysiological recordings

We transferred a single slice to a recording chamber and superfused with ACSF at 4 ml min^−1^. We evoked synaptic response in hippocampal CA1 pyramidal neurons by stimulating Schaffer collateral afferents with bipolar tungsten electrodes located ~50 μm from the pyramidal cell body layer. CA1 pyramidal neurons were visualized by infrared optics on an upright microscope (Zeiss Axioskop 2FS). We performed whole-cell patch-clamp technique at room temperature by using pipettes with a typical resistance of 4–6 MΩ. We recorded baseline responses by stimulating (0.08 min in duration) the slices every 10 s with intensity to evoke a half-maximal EPSP, with patch pipettes containing (in mM): K gluconate 122.5, KCl 17.5, EGTA 0.2, HEPES 10, ATP-Mg 4, Na phosphocreatine 10, GTP 0.3 (pH 7.25, 290 mOsm). In LTP experiments, TBS consisted of 15 bursts of 4 pulses at 100 Hz, delivered to Schaffer collateral afferents at an interburst interval of 200 min. For voltage-clamp recording, pipettes solution containing (in mM): Cs-gluconate 117, CsCl 10, BAPTA 10, CaCl_2_ 1, HEPES 10, ATP-Mg 2, QX-314 10, GTP 0.3 (pH 7.25, osmolarity 290). NMDAR-mediated EPSCs (+60 mV) were pharmacologically isolated by blockade of AMPA receptors and GABAA receptors with bath-applied CNQX (10 µM) or NBQX (10 µM) and bicuculline (10 µM), respectively. Intracellularly administrated Src activating peptide (2 mM), inactive control peptide (2 mM) [29] and Src inhibitory peptide Src40–58 (0.03 mg/ml) [29] were from GenScript. We amplified raw data using a MultiClamp 700B amplifier and a Digidata 1322A acquisition system sampled at 10 KHz and analysed the data with Clampfit 10.6 (Axon Instruments) and Sigmaplot 11 software. In experiments with intracellularly administered Src activating peptide, or the inactive control, synaptic responses were normalized to those in the first 2 min of recording. We performed statistical comparisons of data, presented as mean ± s.e.m., using a one-way analysis of variance with a *post hoc* test.

## Data Availability

We have included the data summarized in the figures in Excel files that are uploaded as supplementary material [73].
